# *In vitro* Activity of Robenidine Analog NCL195 in Combination With Outer Membrane Permeabilizers Against Gram-Negative Bacterial Pathogens and Impact on Systemic Gram-Positive Bacterial Infection in Mice

**DOI:** 10.3389/fmicb.2020.01556

**Published:** 2020-08-04

**Authors:** Hongfei Pi, Hang Thi Nguyen, Henrietta Venter, Alexandra R. Boileau, Lucy Woolford, Sanjay Garg, Stephen W. Page, Cecilia C. Russell, Jennifer R. Baker, Adam McCluskey, Lisa A. O’Donovan, Darren J. Trott, Abiodun D. Ogunniyi

**Affiliations:** ^1^Australian Centre for Antimicrobial Resistance Ecology, School of Animal and Veterinary Sciences, The University of Adelaide, Roseworthy, SA, Australia; ^2^Department of Pharmacology, Toxicology, Internal Medicine and Diagnostics, Faculty of Veterinary Medicine, Vietnam National University of Agriculture, Hanoi, Vietnam; ^3^Health and Biomedical Innovation, Clinical and Health Sciences, University of South Australia, Adelaide, SA, Australia; ^4^School of Animal and Veterinary Sciences, The University of Adelaide, Roseworthy, SA, Australia; ^5^Clinical and Health Sciences, University of South Australia, Adelaide, SA, Australia; ^6^Neoculi Pty. Ltd., Burwood, VIC, Australia; ^7^Chemistry, School of Environmental and Life Sciences, The University of Newcastle, Callaghan, NSW, Australia; ^8^ARC Centre of Excellence in Plant Energy Biology, School of Agriculture, Food & Wine, The University of Adelaide, Urrbrae, SA, Australia

**Keywords:** bacterial sepsis, bioluminescence, cryo-ultramicrotomy, membrane potential, multidrug resistance, NCL195, polymyxin B, transmission electron microscopy

## Abstract

Multidrug-resistant (MDR) pathogens, particularly the ESKAPE group (*Enterococcus faecalis/faecium*, *Staphylococcus aureus*, *Klebsiella pneumoniae*, *Acinetobacter baumannii*, *Pseudomonas aeruginosa*, *Escherichia coli*, and *Enterobacter* spp.), have become a public health threat worldwide. Development of new antimicrobial classes and the use of drugs in combination are potential strategies to treat MDR ESKAPE pathogen infections and promote optimal antimicrobial stewardship. Here, the *in vitro* antimicrobial activity of robenidine analog NCL195 alone or in combination with different concentrations of three outer membrane permeabilizers [ethylenediaminetetraacetic acid (EDTA), polymyxin B nonapeptide (PMBN), and polymyxin B (PMB)] was further evaluated against clinical isolates and reference strains of key Gram-negative bacteria. NCL195 alone was bactericidal against *Neisseria meningitidis* and *Neisseria gonorrhoeae* (MIC/MBC = 32 μg/mL) and demonstrated synergistic activity against *P. aeruginosa*, *E. coli*, *K. pneumoniae*, and *Enterobacter* spp. strains in the presence of subinhibitory concentrations of EDTA, PMBN, or PMB. The additive and/or synergistic effects of NCL195 in combination with EDTA, PMBN, or PMB are promising developments for a new chemical class scaffold to treat Gram-negative infections. Tokuyasu cryo ultramicrotomy was used to visualize the effect of NCL195 on bioluminescent S. aureus membrane morphology. Additionally, NCL195’s favorable pharmacokinetic and pharmacodynamic profile was further explored in *in vivo* safety studies in mice and preliminary efficacy studies against Gram-positive bacteria. Mice administered two doses of NCL195 (50 mg/kg) by the intraperitoneal (IP) route 4 h apart showed no adverse clinical effects and no observable histological effects in major organs. In bioluminescent *Streptococcus pneumoniae* and *S. aureus* murine sepsis challenge models, mice that received two 50 mg/kg doses of NCL195 4 or 6 h apart exhibited significantly reduced bacterial loads and longer survival times than untreated mice. However, further medicinal chemistry and pharmaceutical development to improve potency, solubility, and selectivity is required before efficacy testing in Gram-negative infection models.

## Introduction

Multidrug-resistant (MDR) pathogens, in particular the ESKAPE pathogens (*Enterococcus faecalis/faecium*, *Staphylococcus aureus*, *Klebsiella pneumoniae*, *Acinetobacter baumannii*, *Pseudomonas aeruginosa*, *Escherichia coli*, and *Enterobacter* spp.), are becoming a public health threat in both hospitals and the community. In particular, MDR Gram-negative ESKAPE pathogens significantly impact the standard care of septic patients, owing to high morbidity and mortality rates worldwide (ranging from 30 to 70%) (Giamarellou, 2010; Tamma et al., 2012; Bhattacharya, 2013; Zilberberg et al., 2014; Frieri et al., 2017). Studies have consistently identified MDR Gram-negative ESKAPE pathogens as the main cause of additional hospital-acquired infections, such as respiratory tract, urinary tract, and postsurgical infections (Cardoso et al., 2012; Pendleton et al., 2013; Santajit and Indrawattana, 2016). Moreover, therapeutic options for these infections are becoming more limited, resulting in high health care costs resulting from protracted hospital stays (Ventola, 2015; Centers for Disease Control and Prevention [CDCP], 2019).

In the past, the availability of new antimicrobials kept pace with the evolution of antibiotic-resistant bacteria. However, while there is an increasing trend of multidrug resistance in both Gram-negative and Gram-positive bacteria, there has been a significant global decline of investment into new drug development (Livermore, 2012; Woolhouse and Farrar, 2014). As a result, there are only a limited number of registered alternatives for MDR Gram-negative ESKAPE pathogens and few truly novel classes of antimicrobial agents undergoing preclinical testing in the drug development pipeline (Lepore et al., 2019). Furthermore, within the new classes of antimicrobial agents being developed, most have activity only against Gram-positive pathogens, mainly due to the presence of an outer membrane in Gram-negative bacteria that deters the penetration and retention of antibiotics (Pushpakom et al., 2019; Theuretzbacher et al., 2019).

Previously, our laboratory reported that NCL 195 (4,6-*bis*- (2 -((E) -4-methylbenzylidene)hydrazinyl) pyrimidin - 2 - amine), which was developed as a chemical analog of the anticoccidial drug robenidine, possessed antimicrobial activity against MRSA, vancomycin-resistant enterococci (VRE), and *Streptococcus pneumoniae*. This selectivity is likely attributable to the mode of action of NCL195, which permeabilizes the cytoplasmic membrane of *S. pneumoniae*, VRE, and *S. aureus*, thereby hindering the establishment and maintenance of essential energy sources for cell functioning (Ogunniyi et al., 2017). In the presence of subinhibitory concentrations of outer membrane permeabilizers targeting the outer membrane of bacteria (EDTA, PMBN, and PMB), NCL195 was bactericidal against Gram-negative ESKAPE pathogens reference isolates (*A*. *baumannii*, *E*. *coli*, *K*. *pneumoniae*, and *P*. *aeruginosa*) (MICs of 0.25–8 μg/mL). Meanwhile, in the absence of these outer membrane permeabilizers, NCL195 was also bactericidal against eight *Acinetobacter calcoaceticus* isolates (MIC range from 4 to 32 μg/mL) and one *Acinetobacter anitratus* isolate (MIC = 4 μg/mL) (Abraham et al., 2016; Ogunniyi et al., 2017). Here, we further evaluated the *in vitro* antimicrobial activity of NCL195 in the presence of EDTA, PMBN, or PMB against an expanded panel of Gram-negative pathogens isolated from clinical cases. As a first proof of concept testing of the *in vivo* efficacy of NCL195 against bacterial pathogens, we assessed its *in vivo* safety and efficacy using our established/optimized bioluminescent models of Gram-positive bacterial infection.

## Materials and Methods

### Antimicrobial Agents

**FIGURE 1:**
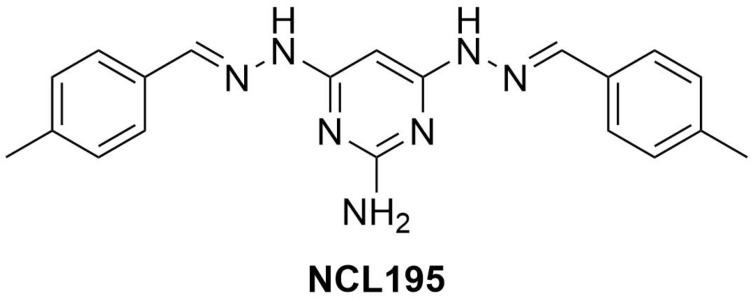
Chemical structure of NCL195 (4,6-*bis*-(2-((E)-4-methylbenzylidene)hydrazinyl)pyrimidin-2-amine).

Analytical grade NCL195 (Figure 1) was synthesized in house at the University of Newcastle as reported previously (Ogunniyi et al., 2017) and stored in a sealed sample container out of direct light at 4°C at the study site at the Infectious Diseases Laboratory, Roseworthy campus, the University of Adelaide. Polymyxin B (PMB) and its derivative, polymyxin B nonapeptide (PMBN), which is devoid of the N-terminal fatty acyl chain and the L-α-γ-diaminobutyric acid residue (and therefore lacks antibacterial activity except against *Pseudomonas* spp.) (Velkov et al., 2010), were purchased from Sigma-Aldrich (NSW, Australia). Stock solutions [containing 25.6 mg/mL of PMBN or PMB in dimethyl sulfoxide (DMSO)] were prepared and stored in 1-mL aliquots at −80°C and defrosted immediately prior to use. Ethylenediaminetetraacetic acid (EDTA, disodium salt) was purchased from Chem-Supply Pty. Ltd., South Australia and was dissolved in Milli-Q water to 200 mM.

### Bacterial Strains

A total of 101 Gram-negative bacteria were collected from government, private, and university diagnostic laboratories throughout Australia. These organisms were speciated using biochemical testing and matrix-assisted laser desorption/ionization–time-of-flight (MALDI-TOF) mass spectrometry (Bruker, Preston, VIC, Australia). The organisms were 18 *A. baumannii* (including two reference strains *A. baumannii* ATCC19606 and *A. baumannii* ATCC 12457), 10 *A. calcoaceticus*, 1 *A. anitratus* and 4 Neisseriae (*N. meningitidis* 423, *N. meningitidis* 424, *N. gonorrhoeae* ATCC 16599, and *N. gonorrhoeae* ATCC 49226). These organisms were kindly provided by Professor Mary Barton (University of South Australia) and the Australian Group on Antimicrobial Resistance (AGAR) for testing the antimicrobial activity of NCL195. Other antimicrobial-resistant bacteria comprising 18 *K. pneumoniae*, 18 *E. coli*, and 19 *P. aeruginosa* human clinical isolates were obtained from the Australian Centre for Antimicrobial Resistance Ecology (ACARE) collection. The following additional 11 Gram-negative reference strains were also used for combination experiments: *E. coli* ATCC 10763, *E. coli* ATCC 25922, *E. coli* ATCC 11229, *Pseudomonas putida* ATCC 17428, *P. aeruginosa* PAO1, *P. aeruginosa* ATCC 27853, *P. aeruginosa* ATCC 33347, *Proteus mirabilis* ATCC 43071, *K. pneumoniae* ATCC 13883, *K. pneumoniae* ATCC 33495, and *K. pneumoniae* ATCC 4352. Bioluminescent *E. coli* (Xen14) (PerkinElmer Inc., Waltham, MA, United States), derived from the parental strain *E. coli* WS2572, and bioluminescent *P. aeruginosa* (Xen41) (PerkinElmer Inc., Waltham, MA, United States), derived from the parental strain *P. aeruginosa* PAO1, were used for the time-dependent killing assays.

For *in vivo* efficacy evaluation of NCL195, bioluminescent *S. aureus* (Xen29), derived from the parental strain *S. aureus* ATCC 12600 (PerkinElmer Inc., Waltham, MA, United States), and bioluminescent *S. pneumoniae*, derived from the parental strain *S. pneumoniae* D39 (D39LUX) (Henken et al., 2010), were used.

### Antimicrobial Susceptibility Testing

Minimum inhibitory concentrations (MICs) were determined in round-bottom 96-well microtiter trays (Sarstedt 82.1582.001), using the modified broth microdilution method recommended by the Clinical and Laboratory Standards Institute [CLSI], 2017. Testing concentrations were as follows: NCL195: 256–0.25 μg/mL; EDTA: 3,800–45 μg/mL; PMBN: 32–0.06 μg/mL; PMB: 32–0.06 μg/mL. Luria–Bertani (LB) broth (Oxoid, VIC, Australia) was used instead of cation-adjusted Mueller–Hinton broth as it was shown previously that robenidine can chelate calcium ions resulting in loss of activity. In addition, twofold serial dilutions of NCL195 were performed in 100% DMSO, with 1 μL added to each well, as NCL compounds have very low solubility in aqueous environments (Abraham et al., 2016). The MICs for ampicillin, gentamicin, and apramycin against each isolate were determined for each test to serve as an internal quality control. The MICs of isolates were determined by visual reading and using an EnSpire Multimode Plate Reader 2300 at *A*_600 nm_. MIC_50_, MIC_90_, and MIC ranges for NCL195, EDTA, PMBN, PMB, or combinations were then determined (Venter, 2019).

### Minimum Bactericidal Concentration Determination

The minimum bactericidal concentration (MBC) of NCL195 alone or in combination with EDTA, PMBN, or PMB was determined against both Gram-positive and Gram-negative bacteria. Briefly, 10-μL aliquots from each duplicate well from the MIC assays (starting from the MIC for each compound) were inoculated onto a sheep blood agar (SBA) plate and incubated at 37°C. Plates were examined at 24 and 48 h and the MBC was recorded as the lowest concentration of each test compound at which a 99.95% colony count reduction was observed on the plate (Clinical and Laboratory Standards Institute [CLSI], 2017).

### Synergy Testing by Checkerboard Microdilution and Dose Reduction Analysis

To assess potential synergistic activity of NCL195, MICs for a range of Gram-negative ATCC strains and clinical isolates of *K. pneumoniae*, *E. coli*, *A. baumannii*, and *P. aeruginosa* were determined in the presence or absence of 23.2–11,400 μg/mL (0.06–30 mM) of EDTA and 0.25–128 μg/mL of PMBN or PMB in a modified standard checkerboard assay as described previously (Hamoud et al., 2015; Khazandi et al., 2019). Briefly, antimicrobial stock solutions were prepared at a concentration of 25.6 mg/mL in DMSO for NCL195, 12.8 mg/mL in Milli-Q water for PMBN and PMB, and 30 mM in Milli-Q water for EDTA. Then, a twofold serial dilution of each antimicrobial stock solution was prepared in its appropriate solvent from wells 12 to 3 (starting from 25.6 to 0.25 mg/mL for NCL195, 12.8 to 0.25 mg/mL for PMBN and PMB, and 30 to 0.06 mM for EDTA). One microliter of each concentration was then added to each well in the challenge plate using an electronic multichannel pipette followed by 89 μL of the LB broth. Ten microliters of bacterial suspension of 1.5 × 10^6^ colony forming units per milliliter (CFU/mL) was added to each well of the plate, which was subsequently incubated at 37°C for 24 h.

The fractional inhibitory concentration index (FICI) describes the results of combination, and was calculated as follows: FICI of combination = FIC A + FIC B. Where FIC A is the MIC of NCL195 in the combination/MIC of NCL195 alone, FIC B is the MIC of EDTA in the combination/MIC of EDTA alone. The results indicate synergism when the corresponding FICI ≤ 0.5, additivity when 0.5 < FICI ≤ 1, indifference when 1 < FICI ≤ 4, and antagonism when the FICI > 4. In this study, the FICI for NCL195 and PMBN against Gram-negative bacteria was calculated to be zero (e.g., 1÷ > 256 = 0), where they did not show any antibacterial activity alone against Gram-negative bacteria at the highest concentration (256 μg/mL).

The dose reduction index (DRI) shows the difference between the effective doses in combination in comparison to its individual dose. DRI was calculated as follows: DRI = MIC of drug alone/MIC of drug in combination. Given that NCL195 and PMBN did not show any antimicrobial activity against the majority of Gram-negative bacteria, the highest concentration of each compound tested against each isolate was used as its MIC alone for calculating the DRI (e.g., MIC of NCL195 alone against *E. coli* 103 was >256 μg/mL and its MIC in combination with PMBN was 1 μg/mL; DRI = 256/1). DRI is very important clinically when the dose reduction is associated with a toxicity reduction without changing efficacy (Eid et al., 2012). Commonly, a DRI higher than 1 is considered beneficial.

### Time-Dependent Killing Assays

Initial time kill assays were performed (in duplicate) for the NCL195 in the presence of PMB against a range of human ESKAPE pathogen reference strains (*E. coli* ATCC 25922, *P. aeruginosa* PAO1, *K. pneumoniae* ATCC 33495, and *A. baumannii* ATCC 19606) as described previously (Clinical and Laboratory Standards Institute [CLSI], 2017) with slight modifications. Briefly, a few colonies of each strain from overnight SBA plates were emulsified in normal saline and adjusted to *A*_600 nm_ = 0.10 (equivalent to approx. 5 × 10^7^ CFU/mL) and the bacterial suspensions were further diluted 1:20 in saline. NCL195 and PMB were serially diluted in 100% DMSO or Milli-Q water at 100 × the final desired concentration and a 100-μL aliquot of appropriate concentrations added to each 10 mL preparation. NCL195 and PMB solutions were prepared in 10-mL volumes at MIC and 2 × MIC concentrations in LB broth. After addition of the inoculum dose to each tube, duplicate cultures were incubated at 37°C, with samples withdrawn at 0, 0.5, 1, 2, 4, 6, 8, and 24 h, serially diluted 10-fold, and plated on SBA overnight at 37°C for bacterial enumeration. The time kill assay was further refined by testing the NCL195-PMB combination on bioluminescent *E. coli* WS2572 (Xen14) and bioluminescent *P. aeruginosa* PAO1 (Xen41). According to CLSI, an antimicrobial agent is considered bactericidal if it causes a ≥3 × log_10_ (99.95%) reduction in CFU/mL after 18–24 h of incubation, and the combination is considered synergistic when it causes a ≥2 × log_10_ reduction in CFU/mL compared with either constituent alone.

### Dual Mechanism of Action of NCL195-PMB Combination

**FIGURE 2:**
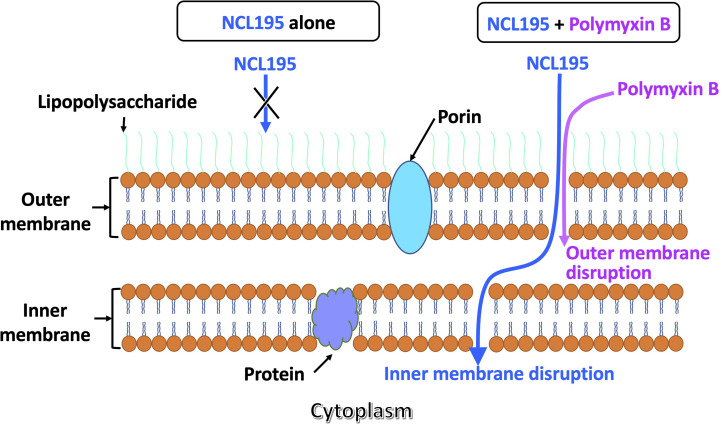
Proposed dual mechanism of action of NCL195 in combination with PMB on Gram-negative bacteria. NCL195 alone is unable to penetrate the outer membrane of Gram-negative bacteria; however, a subinhibitory concentration of PMB can permeabilize the outer membrane and allow penetration of NCL195, resulting in disruption of the inner membrane.

Polymyxin B has been demonstrated to disrupt the outer membrane of Gram-negative bacteria (Lin et al., 2018, 2019; Wang et al., 2020), while NCL195 has been shown to disrupt the inner membrane potential of Gram-positive bacteria (Ogunniyi et al., 2017). Thus, we hypothesized that a combination of PMB and NCL195 will disrupt the outer membrane (PMB), allowing penetration of NCL195 into the inner membrane, as shown in Figure 2.

To prove this hypothesis, the membrane potential of *E. coli* Xen14 cells was measured by fluorescence spectrometry in a LS 55 Fluorescence Spectrometer (PerkinElmer) using the fluorescent membrane potential probe 3,3-diethyloxacarbocyanine iodide [DiOC_2_(3)]. A combination of Gram-negative and Gram-positive membrane potential measurement approaches was used as described previously (Venter et al., 2003; Ogunniyi et al., 2017; Wang et al., 2020). Briefly, *E. coli* Xen14 cells were prepared and resuspended in 50 mM potassium phosphate buffer (pH 7.0) to *A*_600 nm_ = 5. PMB was used at 6.4 μg/mL, while NCL195 was used at either 12.8 or 25.6 μg/mL. The test compounds and/or their combination were incubated with the cells for either 5 or 30 min, after which DiOC_2_(3) was added and the fluorescence monitored until it plateaued. The cells were then energized by the addition of glucose to establish a proton motive force (negative and basic inside the cell). This led to an increase in fluorescence associated with aggregation of the DiOC_2_(3). The membrane potential was then disrupted by the addition of the proton ionophore carbonyl cyanide *m*-chlorophenyl hydrazone (CCCP).

### Transmission Electron Microscopy to Visualize the Effect of NCL195 on Bioluminescent *S. aureus* ATCC12600 (Xen29) Cell Membrane

#### Treatment Preparation

Xen29 was grown on horse blood agar containing 200 μg/mL of kanamycin at 37°C. A single colony was transferred to 10 mL of LB broth in 50-mL Falcon tubes and grown at 37°C under continuous agitation in a reciprocating shaker at 150 rpm. The overnight culture was then diluted 1:30 in 40 mL of LB broth and incubated at 37°C in 50-mL Falcon tubes as previously described until *A*_600 nm_ = 0.1 was obtained. Xen29 was incubated with NCL195 (2 and 4 μg/mL) for 1 h at 37°C, with manual mixing every 10 min. A treatment time of 1 h was chosen based on the time kill kinetic and MIC of NCL195 to ensure Xen29 cells were not killed at the time of harvest. Cells were harvested by centrifugation at 2900 × *g* for 5 min at 4°C. Control cells were harvested at *A*_600 nm_ = 0.1 without the addition of compound. Cells were washed twice in phosphate-buffered saline (PBS) buffer; fixed in 4.0% formaldehyde, 1.25% glutaraldehyde, 0.01 M CaCl_2_, 4% sucrose, and in the presence of 0.075% ruthenium red and 0.075% L-lysine acetate (to stabilize the peptidoglycan layer and aid in locating the bacteria during sectioning); and then stored at 4°C until processing for transmission electron microscopy (TEM). Thereafter, cells were washed twice in PBS and embedded in 12% gelatin. Small gelatin blocks containing bacteria (<1 mm^3^) were cut and infiltrated with 2.3 M sucrose in PBS overnight at 4°C with gentle rocking. Blocks were stored in 2.3 M sucrose at 4°C prior to sectioning.

#### Cryo-Ultramicrotomy and TEM

Blocks were transferred to aluminum cryo-sectioning pins (Leica) and quickly plunge-frozen in liquid nitrogen. Thin cryo-sections (80 nm) were cut at −100°C with an EM-UC6/FC7 cryo-ultramicrotome (Leica) using a cryo-diamond knife (Diatome). Cryo-sections were removed from the knife with 2.3 M sucrose using a wire loop and transferred to formvar/carbon-coated, plasma cleaned, 200-mesh copper EM grids (Proscitech). Grids were stored in an airtight container on sucrose droplets at 4°C. To stain, grids were floated face down on 2% gelatin for 30 min at 37°C before washing in PBS (3 min × 2 min) and staining with 2% uranyloxalicacetate, pH 7 (5 min, room temperature) and methyl cellulose–uranyl acetate pH 4 on ice (10 min). Grids were looped out, drained, and allowed to dry. Samples were imaged with a Tecnai G2 Spirit electron microscope (FEI Company) operated at 100 kV at Adelaide Microscopy, the University of Adelaide, South Australia.

### Ethics Statement

For NCL195 safety and efficacy testing experiments, outbred 5- to 6-week-old male CD1 (Swiss) mice (weighing between 25 and 32 g), obtained from the Laboratory Animal Services breeding facility of the University of Adelaide, were used. Mice had access to food and water *ad libitum* throughout the experiments. The Animal Ethics Committee of the University of Adelaide (approval numbers S-2013-053 and S-2015-151) reviewed and approved all animal experiments. The study was conducted in compliance with the Australian Code of Practice for the Care and Use of Animals for Scientific Purposes (8th edition, 2013) and the South Australian Animal Welfare Act 1985.

### Safety Testing of NCL195 Following Parenteral Administration

We previously determined the pharmacokinetic parameters for NCL195 after a single intraperitoneal administration of 43 mg/kg NCL195 dissolved in 20% (v/v) DMSO in PEG400 with no observed adverse reactions or compound-related side effects (Ogunniyi et al., 2017). It was then assumed that a twofold increase in dose would result in a proportional increase in NCL195 exposure. To test that a two-dose regime would be safe to administer to mice, a safety study was conducted by administering two intraperitoneal doses of NCL195 at either 10 mg/kg or 50 mg/kg 4 h apart to three mice, using two intraperitoneal doses of either PBS or 6 mg/kg daptomycin as controls. Mice were observed for clinical signs and data recorded on a Clinical Record Sheet (CRS) approved by the Animal Ethics Committee of the University of Adelaide. At the conclusion of the experiment, mice were humanely killed and sections of liver, kidneys, spleen, heart, and brain were collected and subjected to histopathological examination.

### Histopathological Examination

Mouse tissues (including liver, spleen, kidneys, heart, and brain) collected from the intraperitoneal safety challenge were fixed in 10% neutral-buffered formalin and processed routinely. The specimens were embedded in paraffin blocks and sections of 4 μm thickness were cut using a microtome. Hematoxylin and eosin staining of the sections was performed and the slides were observed and recorded under light microscopy.

### Efficacy Testing of NCL195 Following Systemic Challenge of Mice With Bioluminescent Gram-Positive Bacteria

For the NCL195 efficacy testing experiments against *S. pneumoniae* challenge, luminescent strain D39 (D39LUX), which emits light constitutively at λ_max_ = 490_nm_ in metabolically active cells, was used, and has been described previously (Henken et al., 2010). Before infection, D39LUX was grown statically in serum broth (10% heat-inactivated horse serum in nutrient broth) at 37°C, 5% CO_2_ to *A*_600 nm_ of 0.16 (equivalent to approx. 5 × 10^7^ CFU/mL). Three groups of mice (*n* = 10 mice per group) were then challenged intraperitoneally with approx. 2.5 × 10^4^ CFU of D39LUX in 100 μL of serum broth. At 12 h postinfection, the conditions of all mice in each group were recorded on a CRS approved by the Animal Ethics Committee of the University of Adelaide. All mice were subjected to bioluminescent imaging in a ventral position on either the Xenogen IVIS 100 system (Xenogen) or the IVIS Lumina XRMS Series III system (Caliper Life Sciences). Immediately after, group 1 mice were administered the drug vehicle only, group 2 received NCL195 at 50 mg/kg i.p., while group 3 received daptomycin at 6 mg/kg i.p. The clinical conditions of all mice were then closely monitored every 2 h, and at 18 h postinfection, all animals in each group were again subjected to bioluminescent imaging. Thereafter, group 1 mice received a second dose of drug vehicle only, while group 2 received a second dose of NCL195 at 50 mg/kg, and mice were further monitored frequently for signs of distress. The daptomycin-treated group did not receive a second dose, as their clinical conditions and bioluminescent imaging data indicated a healthy status. At 24, 30, and 60 h postinfection, mice were further subjected to bioluminescent imaging, and mice that had become moribund or showed any evidence of distress [such as loss of balance, extreme hyperactivity, severe weight loss (>20% body weight), ear temperature falling below 24°C, paralysis, or extreme reluctance or inability to move freely, and/or refusal or inability to eat or drink] were humanely killed by cervical dislocation. A second experiment (*n* = 5) was also performed essentially as described above, but with antimicrobial administration at 8 and 12 h postinfection to assess whether earlier intervention might further prolong the survival times for mice.

For the NCL195 efficacy testing experiments against *S. aureus*, luminescent ATCC12600 strain (Xen29, PerkinElmer) was used, essentially as described previously (Ogunniyi et al., 2018). Briefly, bacteria were grown in LB broth at 37°C to *A*_600 nm_ of 0.5 (equivalent to approx. 1.5 × 10^8^ CFU/mL). Three groups of mice (*n* = 5 mice per group) were challenged intraperitoneally with approx. 2.5 × 10^7^ CFU of Xen29 in 200 μL of PBS containing 3% hog gastric mucin type III (Sigma-Aldrich). At 2 h postinfection, all mice were subjected to bioluminescent imaging in both ventral and dorsal positions on the IVIS Lumina XRMS Series III system. Immediately after, group 1 mice received the drug vehicle only, group 2 received NCL195 at 50 mg/kg i.p., while group 3 received daptomycin at 6 mg/kg i.p. The clinical conditions of all mice were closely monitored, and at 6 h postinfection, all animals in each group were subjected to bioluminescent imaging, after which a second dose of drug vehicle only, NCL195, or daptomycin was administered. Mice were further monitored frequently for signs of distress and those that had become moribund or showed any evidence of distress were humanely killed by cervical dislocation. At 10 and 16 postinfection, living mice were further subjected to bioluminescence imaging. In all experiments, signals were collected from a defined region of interest and total flux intensities (photons/s) analyzed using Living Image Software 2.5 (for IVIS 100) and 4.4 (for Lumina XRMS). Differences in median survival times (time to moribund) for mice between groups were analyzed by the log-rank (Mantel–Cox) tests. Differences in luminescence signals between groups were compared by multiple *t*-tests.

## Results

### NCL195 Alone Shows Antimicrobial Activity Against *N. meningitidis* and *N. gonorrhoeae*

The activity of NCL195 against a range of Gram-negative bacteria (*A. baumannii* ATCC 19606, *A. baumannii* ATCC 12457, *E. coli* ATCC 10763, *E. coli* ATCC 25922, *K. pneumoniae* ATCC 33495, *K. pneumoniae* ATCC 4352, *P. aeruginosa* ATCC 27853, *P. aeruginosa* PAO1, *N. meningitidis* 423, *N. meningitidis* 424, *N. gonorrhoeae* ATCC 16599, and *N. gonorrhoeae* ATCC 49226) was investigated. The results show that NCL195 alone demonstrated antimicrobial activity against the *Neisseria* isolates and type strains tested at 32 μg/mL, but no activity was observed against the other Gram-negative bacteria tested at up to 256 μg/mL (data not shown).

### Combination of NCL195 With EDTA Demonstrates Antimicrobial Activity Against Gram-Negative Pathogen Strains

The modified checkerboard assay was used to assess the combination of NCL195 and EDTA against a range of human Gram-negative pathogen reference strains (*E. coli* ATCC 25922, *E. coli* ATCC 11229, *P. putida* ATCC 17428, *P. aeruginosa* PAO1, *P. aeruginosa* ATCC 27853, *Proteus mirabilis* ATCC 43071, *K. pneumoniae* ATCC 13883, *A. baumannii* ATCC19606, and *A. baumannii* ATCC 12457), and one *A. calcoaceticus* clinical isolate. The results indicate a synergistic interaction of NCL195 and EDTA for *E. coli* ATCC 25922, *E. coli* ATCC 11229, *K. pneumoniae* ATCC 13883, *P. putida* ATCC 17428, *P. aeruginosa* PAO1, and *P. aeruginosa* ATCC 27853. An additive interaction was observed for *P. mirabilis* ATCC 43071, *A. baumannii* ATCC19606, *A. baumannii* ATCC 12457, and the clinical *A. calcoaceticus* isolate (Table 1).

**TABLE 1:** MIC values for NCL195 alone, EDTA alone, and in combination for Gram-negative bacteria reference strains and clinical isolates.

**Isolates**	**MIC (μg/mL; mM concentrations in parentheses)**				**FICI**	**DRI**
	**Single drug**		**Combination**			
	**EDTA**	**NCL195**	**EDTA**	**NCL195**		**EDTA:NCL195**
*E. coli* ATCC 25922	3800 (10)	>256	950 (2.5)	4	0.27^a^	4:64
*E. coli* ATCC 11229	950 (2.5)	>256	228 (0.6)	8	0.27^a^	4:32
*P. putida* ATCC 17428	1900 (5)	>256	950 (2.5)	1.25	0.50^a^	2:204
*P. aeruginosa* PAO1	1900 (5)	>256	475 (1.25)	1.25	0.25^a^	4:204
*P. aeruginosa* ATCC 27853	3800 (10)	>256	1900 (5)	2	0.50^a^	2:128
*P. mirabilis* ATCC 43071	228 (0.6)	>256	228 (0.6)	0.5	1.00^b^	1:512
*K. pneumoniae* ATCC 13883	11 400 (30)	>256	3800 (10)	4	0.35^a^	3:64
*A. baumannii* ATCC19606	380 (1)	>256	190 (0.5)	2	0.51^b^	2:128
*A. baumannii* ATCC 12457	190 (0.5)	>256	95 (0.25)	4	0.52^b^	2:64
*A. calcoaceticus* (a clinical isolate)	228 (0.6)	>256	228 (0.6)	8	1.00^b^	1:32

**Synergy testing by checkerboard microdilution and dose reduction analysis. DRI, dose reduction index.*^*a*^Synergistic effect. ^*b*^Additive effect. The results indicate synergism when the corresponding FICI ≤ *0.5, additivity when 0.5* < *FICI* ≤ *1, indifference when 1* < *FICI* ≤ *4, and antagonism when the FICI* > *4.**

### Combination of NCL195 With Polymyxins (PMBN and PMB) Also Demonstrates Synergistic Activity Against a Range of Gram-Negative ESKAPE Pathogen Reference Strains

The antimicrobial activity of NCL195 in the presence of PMBN was investigated against a range of human ESKAPE pathogen reference strains: 3 *E. coli* strains (*E. coli* ATCC 25922, *E. coli* ATCC 11229, and *E. coli* ATCC 10763), 3 *P. aeruginosa* strains (*P. aeruginosa* ATCC 27853, *P. aeruginosa* ATCC 33347, and *P. aeruginosa* PAO1), 2 *K. pneumoniae* strains (*K. pneumoniae* ATCC 33495 and *K. pneumoniae* ATCC 4352), and 2 *A. baumannii* strains (*A. baumannii* ATCC 19606 and *A. baumannii* ATCC 12457). The combination of NCL195 and PMBN resulted in a synergistic interaction against *E. coli* ATCC 11229, *P. aeruginosa* ATCC 27853, and *K. pneumoniae* ATCC 4352. An additive interaction was recorded against *P. aeruginosa* PAO1 (Table 2). No interaction was detected against the rest of the reference strains tested.

**TABLE 2:** MIC values for NCL195 alone and PMBN alone and in combination for Gram-negative bacteria reference strains and clinical isolates.

**Isolates**	**Single drug (μg/mL)**		**Combination (μg/mL)**			**FICI**	**DRI**
	**PMBN**	**NCL195**		**PMBN**	**NCL195**		**PMBN:NCL195**
*P. aeruginosa* PAO1	1	>256		0.5	4	0.5^a^	2:64
*P. aeruginosa* ATCC 27853	2	>256		0.5	4	0.27^a^	4:64
*E. coli* ATCC 11229	>128	>256		8	32	0.19^a^	16:8
*K. pneumoniae* ATCC 4352	>128	>256		0.125	4	0.03^a^	1024:64

**DRI, dose reduction index.*^*a*^Synergism effect. The results indicate synergism when the corresponding FICI ≤ 0.5, additivity when 0.5 < FICI ≤ 1, indifference when 1 < FICI ≤ 4, and antagonism when the FICI > 4.*

Owing to the high cost of PMBN, we hypothesized that PMB would be a more easily acquired and cost-effective choice of outer membrane permeabilizer with similar efficacy in combination with NCL195 against Gram-negative bacteria. Therefore, the antimicrobial activity of NCL195 and PMB in combination was tested against a larger range of human ESKAPE pathogen isolates (18 *K. pneumoniae* clinical isolates plus *K. pneumoniae* ATCC 33495 and *K. pneumoniae* ATCC 4352, 18 *E. coli* clinical isolates plus *E. coli* ATCC 10763 and *E. coli* ATCC 25922, 16 *A. baumannii* clinical isolates plus *A. baumannii* ATCC 19606 and *A. baumannii* ATCC 12457, 19 *P. aeruginosa* clinical isolates plus *P. aeruginosa* PAO1) (Table 3). The results revealed a synergistic interaction of NCL195 and PMB in combination against all Gram-negative isolates tested (reducing the MIC of NCL195 by 64- to 1,024-fold against all Gram-negative species tested).

**TABLE 3:** MIC range and MIC_50_, MIC_90_, and DRI values for NCL195 alone, PMB alone, and in combination against 20 *K. pneumoniae*, 20 *E. coli*, 18 *A. baumannii*, and 20 *P. aeruginosa* clinical isolates.

**Isolates**	**Value**	**Antimicrobial concentration (μg/mL)**				**Combination Effect**	**DRI**	
		**Single drug**		**Combination**				
		**PMB**	**NCL195**	**PMB**	**NCL195**		**PMB**	**NCL195**
*K. pneumoniae* (*n* = 20)	MIC range	0.125–1	>256	0.06–0.5	0.25–4	Synergism	2–4	64–1024
	MIC_50_	0.5	>256	0.125	2		4	128
	MIC_90_	1	>256	0.25	4		2	64
*E. coli* (*n* = 20)	MIC range	0.125–1	>256	0.06–0.125	0.25–2	Synergism	2–8	128–1024
	MIC_50_	0.5	>256	0.06	1		8	256
	MIC_90_	0.5	>256	0.125	2		4	128
*A. baumannii* (*n* = 18)	MIC range	0.5–1	>256	0.125	0.5–4	Synergism	4–8	64–512
	MIC_50_	1	>256	0.125	2		8	128
	MIC_90_	1	>256	0.125	2		4	128
*P. aeruginosa* (*n* = 20)	MIC range	0.25–1	>256	0.06–0.25	1–2	Synergism	4–8	128–256
	MIC_50_	0.5	>256	0.125	1		4	256
	MIC_90_	0.5	>256	0.125	2		4	128

### Time Kill Kinetics of the Combination of NCL195 and PMB Shows Bactericidal Antimicrobial Activity Against *E. coli* and *P. aeruginosa* Reference Strains

**FIGURE 3:**
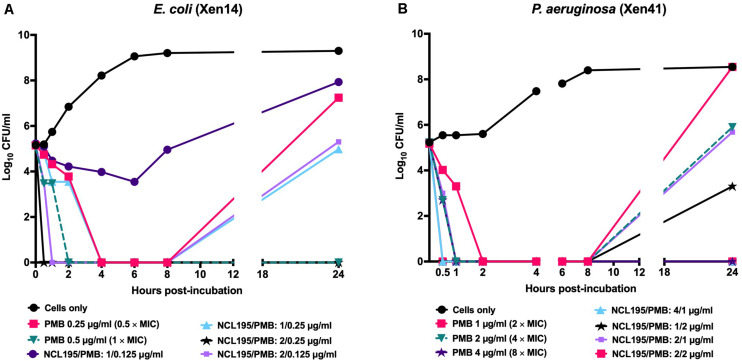
Time kill curves of NCL195 and PMB combinations against **(A)** bioluminescent *E. coli* (Xen14): NCL195/PMB: 1/0.125 μg/mL; NCL195/PMB: 2/0.125 μg/mL; NCL195/PMB: 1/0.25 μg/mL; NCL195/PMB: 2/0.125 μg/mL and **(B)** bioluminescent *P. aeruginosa* (Xen41): NCL195/PMB: 2/1 μg/mL; NCL195/PMB: 4/1 μg/mL; NCL195/PMB: 1/2 μg/mL; NCL195/PMB: 2/2 μg/mL.

Initial time kill kinetics of the NCL195-PMB combination against *E. coli* ATCC 25922, *P. aeruginosa* PAO1, *K. pneumoniae* ATCC 33495, and *A. baumannii* ATCC 19606 showed rapid, time-dependent killing of the bacteria (Supplementary Figure S1). The assay was further refined using bioluminescent *E. coli* WS2572 (Xen14) and bioluminescent *P. aeruginosa* PAO1 (Xen41). For Xen14, NCL195/PMB at 1/0.125 μg/mL showed no antimicrobial activity; NCL195/PMB at 2/0.125 μg/mL reduced the CFU/mL by 5 × log_10_ by 1 h post-treatment but then growth started again after 8 h; NCL195/PMB at 1/0.25 μg/mL reduced the CFU/mL by 5 × log_10_ by 6 h post-treatment but then growth started again after 8 h; NCL195/PMB at 2/0.25 μg/mL reduced the CFU/mL by 5 × log_10_ by 30 min post-treatment and totally cleared the bacterial growth (Figure 3A). For Xen41, NCL195/PMB at 2/1 μg/mL reduced the CFU/ml by 5 × log_10_ within 1 h of treatment but then growth started again after 8 h; NCL195/PMB at 4/1 μg/mL reduced the CFU/mL by 5 × log_10_ by 30 min post-treatment and totally cleared the bacterial growth; NCL195/PMB at 1/2 μg/mL reduced the CFU/ml by 5 × log_10_ by 30 min post-treatment but then growth started again after 8 h; NCL195/PMB at 2/2 μg/mL reduced the CFU/mL by 5 × log_10_ by 30 min post-treatment and totally cleared the bacterial growth (Figure 3B).

### Combination of PMB and NCL195 Disrupts the Inner and Outer Membrane Potential of *E. coli*

**FIGURE 4:**
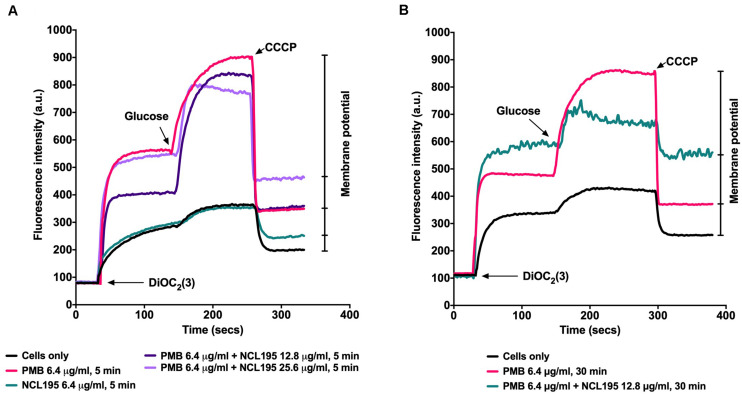
Dual mechanism of action of NCL195-PMB combination. *E. coli* Xen14 cell suspensions were exposed to 6.4 μg/mL of NCL195 or 6.4 μg/mL of PMB alone or a combination of NCL195 and PMB at the indicated concentrations for either 5 min **(A)** or 30 min **(B)** after which DiOC_2_(3) was added and the fluorescence monitored until it plateaued. Cells were then reenergized with 0.5% glucose and the establishment of a membrane potential was measured as an increase in fluorescence until it plateaued. The membrane potential was then disrupted by the addition of the proton ionophore (CCCP).

We tested the hypothesis that a combination of PMB and NCL195 will disrupt the outer membrane (PMB), allowing penetration of NCL195 into the inner membrane of Gram-negative bacteria by measuring the membrane potential of *E. coli* Xen14 cells using fluorescence spectrometry as described in Section “Materials and Methods” (see section “Dual Mechanism of Action of NCL195-PMB Combination”). We showed that preincubation of the cells with PMB at 6.4 μg/mL permeabilized the outer membrane so that greater quantities of the DIOC_2_(3) could gain entry into the cells without affecting the inner membrane (Figures 4A,B). Preincubation of the cells with the NCL195 + PMB combination resulted in a time-dependent and NCL195 concentration-dependent disruption of the inner membrane potential, clearly demonstrating the dual mechanism of action of NCL195-PMB combination.

### NCL195 Exerts Its Antibacterial Action on the Cell Membrane of *S. aureus*

**FIGURE 5:**
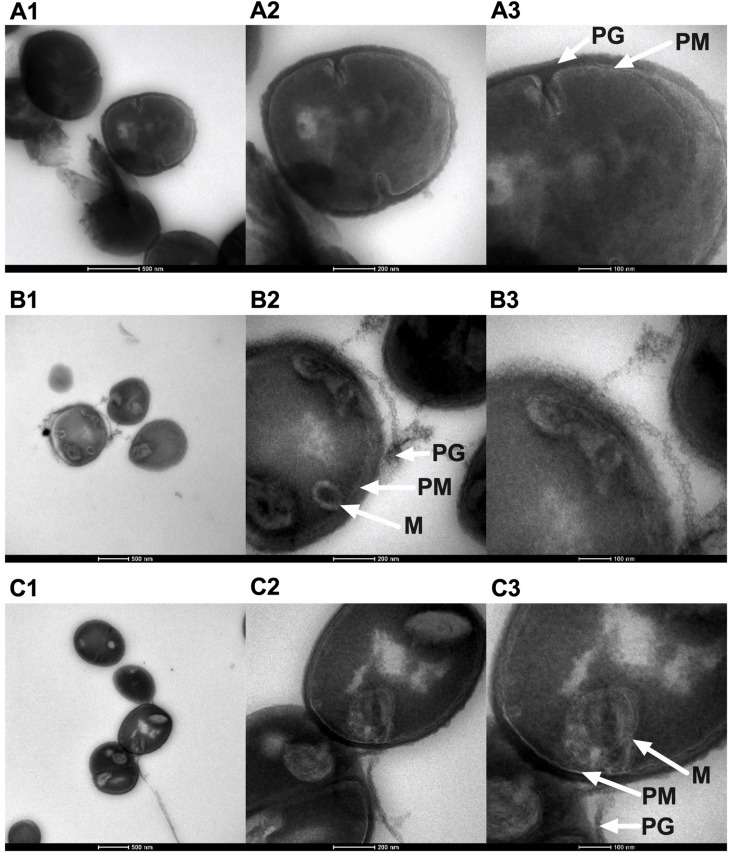
Morphology of bioluminescent *S. aureus* Xen29 prepared using Tokuyasu cryo-ultramicrotomy and visualized by TEM. **(A1–A3)** Xen29 without treatment. **(B1–B3)** Xen29 treated with NCL195 at 2 μg/mL. **(C1–C3)** Xen 29 treated with NCL195 at 4 μg/mL. Arrows show peptidoglycan layer (PG), plasma membrane (PM) and mesosome-like structures (M). NCL195 treatment of Xen29 (*A*_600 nm_ = 0.1) at either 2 μg/mL (1 × MIC) or 4 μg/mL (2 × MIC) for 1 h causes detachment of the cell wall from the cell membrane. Scale bars: **(A1,B1,C1)**: 500 nm; **(A2,B2,C2)**: 200 nm; **(A3,B3,C3)**: 100 nm.

We previously showed that NCL195 acts on the cell membranes of *S. pneumoniae* (MIC range of 2–8 μg/ml) and *S. aureus* (MIC range of 1–2 μg/mL) via disruption of the membrane potential (Ogunniyi et al., 2017) and demonstrated changes in the appearance and thickness of the NCL195-treated cell membrane for *S. pneumoniae* by TEM, but this was not carried out for *S. aureus*. In this study, we used Tokuyasu ultrathin cryo-sections to visualize the effect of NCL195 on bioluminescent *S. aureus* Xen29 membrane morphology. TEM images showed optimal structural preservation with clear delineation of the plasma membrane and cell wall peptidoglycan layer. Samples treated with NCL195 clearly show perturbation of the membrane consistent with its membrane-acting property. In the treated sample, mesosome-like membrane structures were also observed (Figure 5).

### NCL195 Shows Systemic Safety in Mice

**FIGURE 6:**
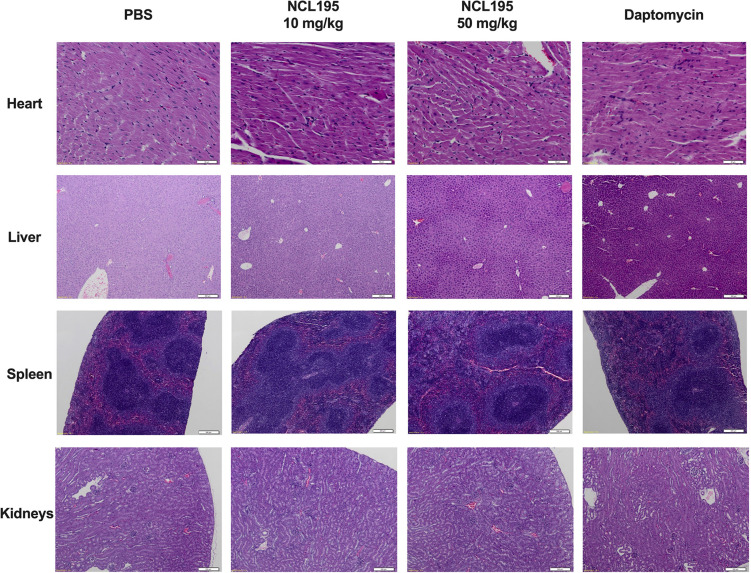
Selected histological images of heart, liver, spleen, and kidneys from treated and control mice. No morphological abnormalities or changes were observed in mice subject to NCL195 (10 mg/kg), NCL195 (50 mg/kg), or daptomycin compared with PBS. Scale bars: heart: 50 μm; liver, spleen, and kidneys: 200 μm.

There were no observable histopathological changes in the heart, liver, spleen, kidneys, or brain (not shown) of any of the mice in the treated groups (NCL195 10 mg/kg, NCL195 50 mg/kg, and daptomycin) compared to the control group treated with PBS (Figure 6).

### Treatment of Mice With NCL195 Reduces *S. pneumoniae* and *S. aureus* Populations and Significantly Prolongs Survival Times

**FIGURE 7:**
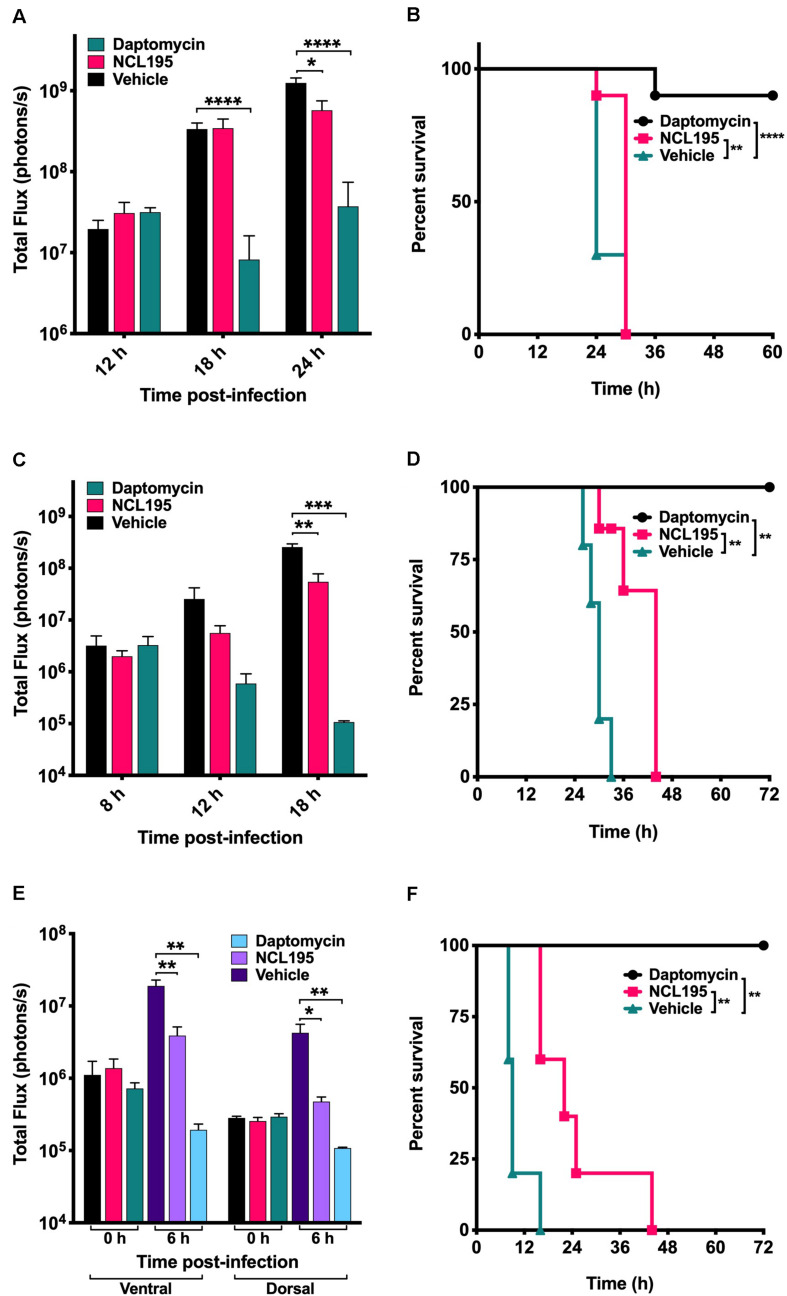
Luminescence signal comparisons between groups of CD1 mice challenged intraperitoneally with *S. pneumoniae* (D39LUX) treated 6 h apart **(A,B)** or treated 4 h apart **(C,D)**, and bioluminescent *S. aureus* ATCC 12600 (Xen29) treated 4 h apart **(E,F)**. Mice were subjected to bioluminescent imaging on an IVIS Lumina XRMS Series III system. *p < 0.05; **p < 0.01; ***p < 0.001; ****p < 0.0001.

**FIGURE 8:**
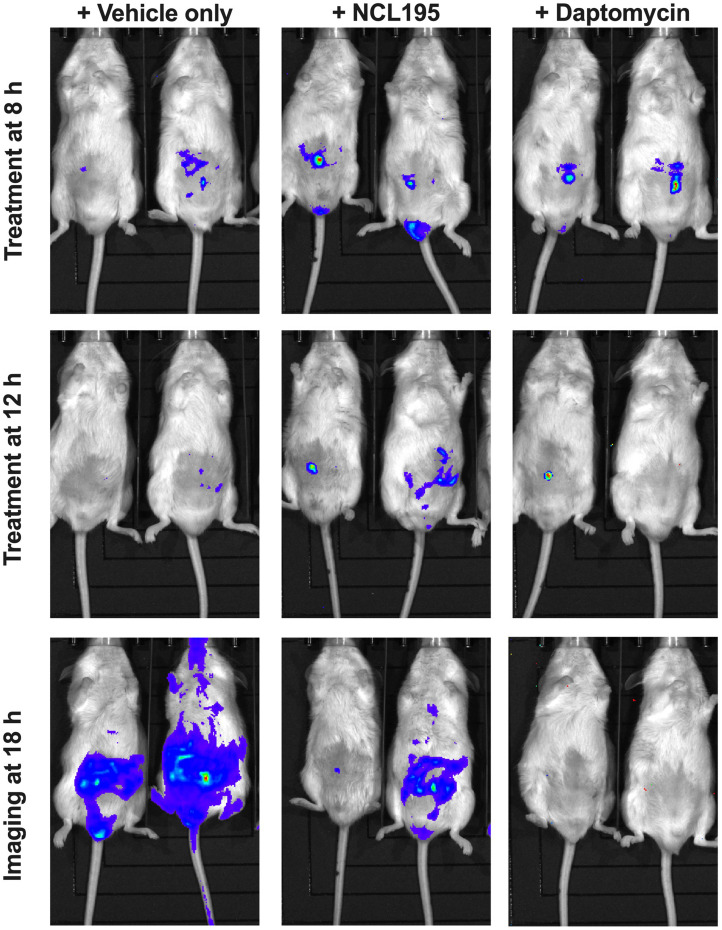
Ventral images of representative CD1 mice challenged with approx. 1 × 10^7^ CFU of bioluminescent *S. pneumoniae* (D39LUX). Mice were subjected to bioluminescence imaging on an IVIS Lumina XRMS Series III system at the indicated times.

**FIGURE 9:**
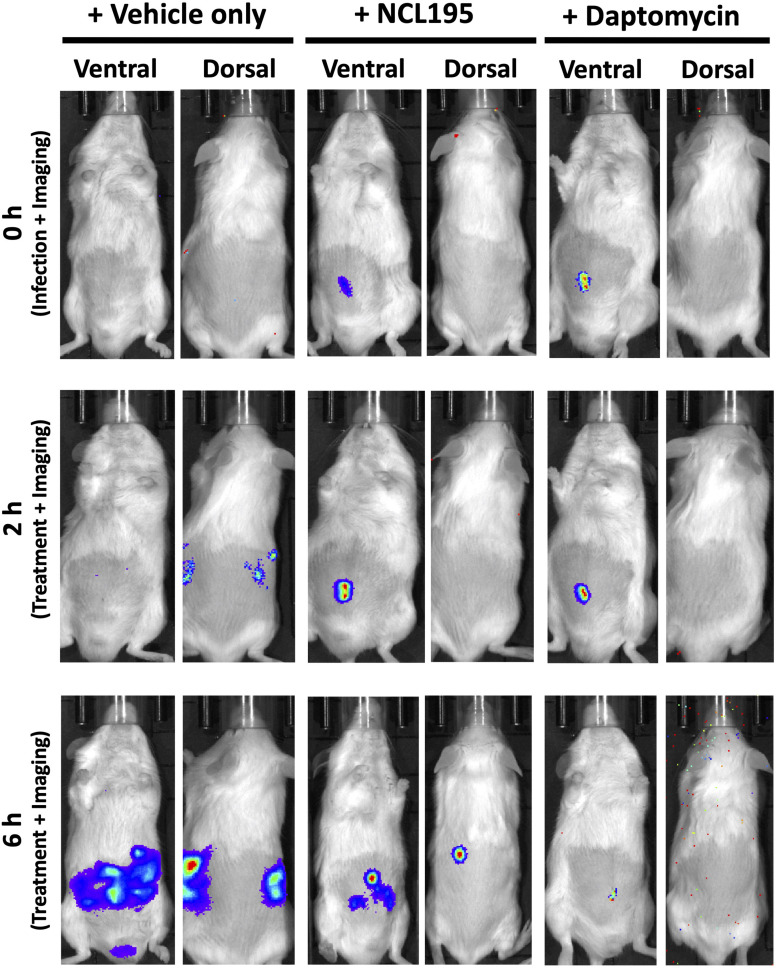
Ventral and dorsal images of representative CD1 mice challenged with approx. 1 × 10^7^ CFU of bioluminescent *S. aureus* ATCC 12600 (Xen29). Mice were subjected to bioluminescence imaging on an IVIS Lumina XRMS Series III system at the indicated times.

We evaluated the potential of NCL195 as a therapeutic drug against systemic *S. pneumoniae* infection, using a well-characterized luminescent strain (D39LUX) (Henken et al., 2010) in an intraperitoneal sepsis challenge model. We found that at 24 h postinfection, two doses of NCL195 at 50 mg/kg i.p. (administered at 12 and 18 h postinfection) resulted in a statistically significant reduction in *S. pneumoniae* populations (*p* = 0.018, multiple *t*-tests; Figure 7A) and a concomitant significant increase in median survival time compared to the vehicle only control in this model (*p* = 0.008; Mantel–Cox test; Figure 7B). In a repeat experiment, intraperitoneal administration of two doses of NCL195 at 50 mg/kg at 8 and 12 h postinfection resulted in an earlier and statistically significant reduction in *S. pneumoniae* populations at 18 h postinfection (*p* = 0.002, multiple *t*-tests; Figures 7C, 8) as well as significant increase in median survival time compared to the vehicle only control (*p* = 0.003; Mantel–Cox test; Figure 7D). Furthermore, intraperitoneal administration of two doses of NCL195 at 50 mg/kg at 2 and 6 h postinfection resulted in a statistically significant reduction in *S. aureus* populations in the blood and mouse kidneys at 6 h postinfection (*p* = 0.006 and 0.021, respectively, multiple *t*-tests; Figures 7E, 9) as well as a significant increase in median survival time compared to the vehicle only control (*p* = 0.009; Mantel–Cox test; Figure 7F). No adverse effects attributable to drug treatment were observed in the three rodent studies.

## Discussion

There is an urgent need to develop new broad-spectrum antimicrobials with activity against pan resistant ESKAPE pathogens, particularly Gram-negative members, with a view to overcoming the permeability barrier of the Gram-negative outer membrane (Willyard, 2017; Venter, 2019; World Health Organization [WHO], 2019). Previously, we reported that NCL195 showed potential as a new drug scaffold for the treatment of infections caused by Gram-positive bacteria on the basis of its favorable pharmacokinetic and pharmacodynamics profile compared to the parent compound robenidine (Ogunniyi et al., 2017). Here, we extended our analyses by assessing *in vitro* efficacy of NCL195 against a range of clinical human Gram-negative pathogens in the presence or absence of subinhibitory concentrations of EDTA, PMBN, or PMB and by conducting preliminary *in vivo* safety and efficacy studies against two prominent Gram-positive bacteria. This study had three major findings. First, NCL195 demonstrated antimicrobial activity against a variety of Gram-negative ESKAPE pathogens in the presence of outer membrane permeabilizers (EDTA, PMB, and PMBN). In particular, combination of NCL195 with PMB showed the best synergistic activity against all the bacteria tested. Second, NCL195 showed systemic safety in mice without any apparent morphological effects on the major organs examined. However, as this study has only examined histomorphology but not clinical chemistry and hematology, sublethal effects not resulting in morphological changes of tissues cannot be excluded. Third, systemic treatment of mice with NCL195 reduced bioluminescent *S. pneumoniae* and *S. aureus* populations *in vivo* and significantly prolonged survival times.

We demonstrated that the combination of EDTA with NCL195 resulted in a synergistic interaction when tested against *E. coli*, *K. pneumoniae*, *P. putida*, and *P. aeruginosa* type and clinical strains. In combination, NCL195 MICs ranged from 0.5 to 8 μg/mL in the presence of EDTA concentrations ranging from 190 to 11,400 μg/mL. However, the lowest dose of EDTA to cause a toxic effect in animals is reported to be 750 mg/kg/day (Lanigan and Yamarik, 2002). In addition, oral exposure to EDTA produced adverse reproductive and developmental effects in animals (Lanigan and Yamarik, 2002). Therefore, EDTA in synergistic combination with NCL195 is not considered to be a viable option for administration by the oral, intravenous, and intramuscular routes in humans and animals.

In combination with PMBN (which alone had no antimicrobial activity against the tested Gram-negative bacteria except for *P. aeruginosa*), NCL195 exhibited a synergistic interaction against less than half of the tested Gram-negative bacteria, with the NCL195 MICs ranging from 4 to 32 μg/mL. The lack of activity of PMBN against Gram-negative bacteria has been attributed to the absence of N-terminal fatty acyl chain present in PMB and highlights the importance of both the electrostatic and hydrophobic interactions for the mechanism of PMB action (Velkov et al., 2010). Our results demonstrated that the combination of PMBN with NCL195 is not ideal for the treatment of human systemic Gram-negative bacterial infections. PMB, with antimicrobial activity against Gram-negative bacteria, could be a better and less expensive choice to be used in combination with NCL195 to treat Gram-negative bacteria, reducing the required concentrations for both drugs, and resulting in better cytotoxicity profiles (Deris et al., 2014; Brown and Dawson, 2017; Lin et al., 2018, 2019).

Previous studies investigating the metabolic pathways impacted by PMB alone or in combination with enrofloxacin was carried out to discover the mechanism of action of the combination against Gram-negative bacteria (Lin et al., 2019). It was found that a large number of metabolites associated with fatty acid and lipid metabolism pathways were significantly perturbed following treatment of *P. aeruginosa* with subinhibitory concentration of PMB alone or in combination with enrofloxacin at 1 and 4 h, consistent with the mode of action of PMB in the disruption of the bacterial outer membrane (Lin et al., 2018, 2019). These pathways play an important role in the DNA repair process; therefore PMB alone can disrupt these processes to prevent the self-repair mechanisms in the bacteria. On the other hand, we previously showed that NCL195 disrupts the inner membrane potential of Gram-positive bacteria and inhibits DNA and RNA synthesis, thereby hindering the establishment and maintenance of essential energy sources for cell functioning (Ogunniyi et al., 2017). Thus, we hypothesized that a combination of PMB and NCL195 will disrupt the outer membrane (PMB), hence allowing penetration of NCL195 to the inner membrane, where it will exert its mechanism of action in preventing DNA/RNA synthesis and altering energy metabolism and cell envelope biogenesis. The combined action of PMB and NCL195 was expected to be synergistic, resulting in the antimicrobial activity of the combination against Gram-negative bacteria, as was demonstrated in the MIC results reported here. The proposed dual mechanism of action of the NCL195-PMB combination was proven in this study by demonstrating a time-dependent and NCL195 concentration-dependent disruption of the inner membrane potential of *E. coli* cells.

Further screening of a broader range of clinical Gram-negative bacteria isolated from humans (18 *K. pneumoniae*, 18 *E. coli*, 16 *A. baumannii*, and 19 *P. aeruginosa*) proved our hypothesis that in the presence of PMB, NCL195 could inhibit the growth of the ESKAPE pathogens (MICs ranging from 0.25 to 4 μg/mL) (Ogunniyi et al., 2017). The current results show that the MIC of NCL195 was significantly reduced 32- to 512-fold, and the MIC of PMB was reduced 2- to 8-fold. Although it was previously reported that PMB is toxic for humans at a concentration of 4 μg/mL, the lowest concentration required in combination with NCL195 ranged from 0.0625 to 0.25 μg/mL, which is 64- to 16-fold lower than its cytotoxic dose (Roberts et al., 2015; Ahmed et al., 2017). Overall, PMB was the best choice among the three outer membrane permeabilizers, due to the subinhibitory activity against all Gram-negative species, as well as lower MICs and less toxicity potential.

We previously used fluorescence-based membrane potential measurements to show that NCL195 and other NCL compounds permeabilize the cytoplasmic membrane of *S. pneumoniae* and *S. aureus*, and hence hinder the establishment and maintenance of essential energy sources for cell functioning (Ogunniyi et al., 2017). In this study, we extended our investigation on the effects of NCL195 on bioluminescent *S. aureus* morphology prepared using Tokuyasu cryo-ultramicrotomy. TEM images show membrane morphology changes and the presence of mesosome-like membrane structures in NCL195-treated but not in untreated bacteria. The presence of mesosomes is possibly a consequence of cell membrane perturbation through disruption of the membrane potential, consistent with the effects of other antibiotic treatments reported by other workers (Friedrich et al., 2000; Li et al., 2008; Morita et al., 2015; Gao et al., 2019).

We also found that two intraperitoneal administrations of NCL195 at 50 mg/kg at 4 h apart was safe in mice without any demonstrable clinical signs or observable morphological effects on the main organs examined. Given these findings, there is a possibility of using PMB in combination with NCL195 for human use after appropriate testing in animal models of infection.

Based on the above, we evaluated the potential of NCL195 as a therapeutic drug against acute systemic *S. pneumoniae* or *S. aureus* infection, using luminescent derivatives of highly virulent pneumococcal strain (D39LUX) and *S. aureus* ATCC12600 (Xen29) in an intraperitoneal challenge infection model. Our results show that two 50 mg/kg i.p. doses of NCL195 resulted in a statistically significant reduction in both *S. pneumoniae* or *S. aureus* populations and prolonged survival times compared to the vehicle-only control. These results suggest that while plasma binding increases the MIC for NCL195 by fourfold against *S. aureus in vitro*, it does not appear to affect its *in vivo* activity, as judged by the statistically significant increase in the median survival times of mice that received NCL195 compared to the vehicle only group. While NCL195 did not achieve the level of potency seen with daptomycin, we have shown that no resistance developed above the MIC for *S. aureus* over 24 serial passages, whereas resistance to daptomycin developed by day 5, and increased up to 8 × MIC by day 12 of the serial passage (Ogunniyi et al., 2017). A low propensity to select resistance is a desirable characteristic for further exploration of NCL195 as a novel antimicrobial class to treat acute bacterial infections in humans. Furthermore, it is reported that daptomycin, which is active against resistant Gram-positive bacteria, does not have antimicrobial activity against most Gram-negative bacteria, even in combination with antimicrobials and outer membrane permeabilizers (Phee et al., 2013). Together, our findings demonstrate that the new antibacterial class represented by NCL195 could provide promising new scaffolds for further pharmaceutical and medicinal chemistry development. Further chemical diversification of the NCL195 scaffold is desirable to increase solubility and reduce plasma binding and potential toxicity, as well as improve potency against the antimicrobial resistant pathogens currently listed as most urgent priority for antibacterial drug discovery and development (Willyard, 2017; World Health Organization [WHO], 2019).

## Data Availability Statement

All datasets generated for this study are included in the article/Supplementary Material.

## Ethics Statement

The animal study was reviewed and approved by The Animal Ethics Committee of The University of Adelaide (approval numbers S-2013-053 and S-2015-151). The study was conducted in compliance with the Australian Code of Practice for the Care and Use of Animals for Scientific Purposes (8th Edition 2013) and the South Australian Animal Welfare Act 1985.

## Author Contributions

HP, HN, HV, SP, AM, LO’D, DT, and AO: conceptualization. HP, HN, AB, LW, LO’D, and AO: data curation and methodology. HP, HN, HV, AB, LW, SG, SP, AM, LO’D, DT, and AO: formal analysis. HV, SG, SP, AM, DT, and AO: funding acquisition. HP, HN, AB, LW, SP, LO’D, DT, and AO: investigation. CR, JB, and AM: NCL195 synthesis. SG, SP, AM, DT, and AO: project administration. HV, LW, SG, SP, AM, and DT: resources. HV, SG, AM, DT, and AO: supervision. HV, SP, DT, and AO: validation. HP, HN, and AO: writing—original draft. HP, HN, HV, AB, LW, SG, SP, CR, AM, LO’D, DT, and AO: writing—review and editing. All authors contributed to the article and approved the submitted version.

## Conflict of Interest

SP is a director of Neoculi Pty. Ltd. The remaining authors declare that the research was conducted in the absence of any commercial or financial relationships that could be construed as a potential conflict of interest.

## References

[B1] Abraham R. J. Stevens A. J. Young K. A. Russell C. Qvist A. Khazandi M. (2016). Robenidine analogues as Gram-positive antibacterial agents. *J. Med. Chem.* 59 2126–2138. 10.1021/acs.jmedchem.5b01797 26765953

[B2] Ahmed M. U. Velkov T. Lin Y.-W. Yun B. Nowell C. J. Zhou F. (2017). Potential toxicity of polymyxins in human lung epithelial cells. *Antimicrob. Agents Chemother.* 61:e02690-16. 10.1128/AAC.02690-16 PMC5444173 28416543

[B3] Bhattacharya S. (2013). Early diagnosis of resistant pathogens: how can it improve antimicrobial treatment? *Virulence* 4 172–184. 10.4161/viru.23326 23302786 PMC3654618

[B4] Brown P. Dawson M. J. (2017). Development of new polymyxin derivatives for multi-drug resistant Gram-negative infections. *J. Antibiot.* 70 386–394. 10.1038/ja.2016.146 28074057

[B5] Cardoso T. Ribeiro O. Aragão I. C. Costa-Pereira A. Sarmento A. E. (2012). Additional risk factors for infection by multidrug-resistant pathogens in healthcare-associated infection: a large cohort study. *BMC Infect. Dis.* 12:375. 10.1186/1471-2334-12-375 23267668 PMC3566942

[B6] Centers for Disease Control and Prevention [CDCP] (2019). *Antibiotic Resistance Threats in the United States, 2019.* Atlanta, GA: U.S. Department of Health and Human Services, CDC.

[B7] Clinical and Laboratory Standards Institute [CLSI] (2017). *Performance Standards for Antimicrobial Susceptibility Testing. 27th ed. CLSI standard M100.* Wayne, PA: CLSI.

[B8] Deris Z. Z. Swarbrick J. D. Roberts K. D. Azad M. A. Akter J. Horne A. S. (2014). Probing the penetration of antimicrobial polymyxin lipopeptides into Gram-negative bacteria. *Bioconjug. Chem.* 25 750–760. 10.1021/bc500094d 24635310 PMC3993906

[B9] Eid S. Y. El-Readi M. Z. Wink M. (2012). Synergism of three-drug combinations of sanguinarine and other plant secondary metabolites with digitonin and doxorubicin in multi-drug resistant cancer cells. *Phytomedicine* 19 1288–1297. 10.1016/j.phymed.2012.08.010 23146422

[B10] Friedrich C. L. Moyles D. Beveridge T. J. Hancock R. E. (2000). Antibacterial action of structurally diverse cationic peptides on Gram-positive bacteria. *Antimicrob. Agents Chemother.* 44 2086–2092. 10.1128/aac.44.8.2086-2092.2000 10898680 PMC90018

[B11] Frieri M. Kumar K. Boutin A. (2017). Antibiotic resistance. *J. Infect. Public Health* 10 369–378. 27616769 10.1016/j.jiph.2016.08.007

[B12] Gao Z. Van Nostrand J. D. Zhou J. Zhong W. Chen K. Guo J. (2019). Anti-Listeria activities of linalool and its mechanism revealed by comparative transcriptome analysis. *Front. Microbiol.* 10:2947. 10.3389/fmicb.2019.02947 31921091 PMC6938037

[B13] Giamarellou H. (2010). Multidrug-resistant Gram-negative bacteria: how to treat and for how long. *Int. J. Antimicrob. Agents* 36 (Suppl. 2), S50–S54. 21129924 10.1016/j.ijantimicag.2010.11.014

[B14] Hamoud R. Reichling J. Wink M. (2015). Synergistic antibacterial activity of the combination of the alkaloid sanguinarine with EDTA and the antibiotic streptomycin against multidrug resistant bacteria. *J. Pharm. Pharmacol.* 67 264–273. 10.1111/jphp.12326 25495516

[B15] Henken S. Bohling J. Ogunniyi A. D. Paton J. C. Salisbury V. C. Welte T. (2010). Evaluation of biophotonic imaging to estimate bacterial burden in mice infected with highly virulent compared to less virulent Streptococcus pneumoniae serotypes. *Antimicrob. Agents Chemother.* 54 3155–3160. 10.1128/aac.00310-10 20530224 PMC2916300

[B16] Khazandi M. Pi H. Chan W. Y. Ogunniyi A. D. Sim J. X. F. Venter H. (2019). In vitro antimicrobial activity of robenidine, ethylenediaminetetraacetic acid and polymyxin B nonapeptide against important human and veterinary pathogens. *Front. Microbiol.* 10:837. 10.3389/fmicb.2019.00837 31105656 PMC6494957

[B17] Lanigan R. S. Yamarik T. A. (2002). Final report on the safety assessment of EDTA, calcium disodium EDTA, diammonium EDTA, dipotassium EDTA, disodium EDTA, TEA-EDTA, tetrasodium EDTA, tripotassium EDTA, trisodium EDTA, HEDTA, and trisodium HEDTA. *Int. J. Toxicol.* 21 (Suppl. 2), 95–142. 10.1080/10915810290096522 12396676

[B18] Lepore C. Silver L. Theuretzbacher U. Thomas J. Visi D. (2019). The small-molecule antibiotics pipeline: 2014-2018. *Nat. Rev. Drug Discov.* 18:739. 10.1038/d41573-019-00130-8 31570838

[B19] Li X. Feng H. Q. Pang X. Y. Li H. Y. (2008). Mesosome formation is accompanied by hydrogen peroxide accumulation in bacteria during the rifampicin effect. *Mol. Cell. Biochem.* 311 241–247. 10.1007/s11010-007-9690-4 18163201

[B20] Lin Y.-W. Han M.-L. Zhao J. Zhu Y. Rao G. Forrest A. (2019). Synergistic combination of polymyxin B and enrofloxacin induced metabolic perturbations in extensive drug-resistant *Pseudomonas aeruginosa*. *Front. Pharmacol.* 10:1146. 10.3389/fphar.2019.01146 31632279 PMC6785843

[B21] Lin Y.-W. Heidi H. Y. Zhao J. Han M.-L. Zhu Y. Akter J. (2018). Polymyxin B in combination with enrofloxacin exerts synergistic killing against extensively drug-resistant *Pseudomonas aeruginosa*. *Antimicrob. Agents Chemother.* 62:e0028-18. 10.1128/AAC.00028-18 PMC5971615 29632010

[B22] Livermore D. M. (2012). Current epidemiology and growing resistance of Gram-negative pathogens. *Korean J. Intern. Med.* 27:128. 10.3904/kjim.2012.27.2.128 22707882 PMC3372794

[B23] Morita D. Sawada H. Ogawa W. Miyachi H. Kuroda T. (2015). Riccardin C derivatives cause cell leakage in *Staphylococcus aureus*. *Biochim. Biophys. Acta* 1848 2057–2064. 10.1016/j.bbamem.2015.05.008 26003535

[B24] Ogunniyi A. D. Khazandi M. Stevens A. J. Sims S. K. Page S. W. Garg S. (2017). Evaluation of robenidine analog NCL195 as a novel broad-spectrum antibacterial agent. *PLoS One* 12:e0183457. 10.1371/journal.pone.0183457 28873428 PMC5584945

[B25] Ogunniyi A. D. Kopecki Z. Hickey E. E. Khazandi M. Peel E. Belov K. (2018). Bioluminescent murine models of bacterial sepsis and scald wound infections for antimicrobial efficacy testing. *PLoS One* 13:e0200195. 10.1371/journal.pone.0200195 30011298 PMC6047774

[B26] Pendleton J. N. Gorman S. P. Gilmore B. F. (2013). Clinical relevance of the ESKAPE pathogens. *Expert Rev. Anti Infect. Ther.* 11 297–308. 10.1586/eri.13.12 23458769

[B27] Phee L. Hornsey M. Wareham D. W. (2013). In vitro activity of daptomycin in combination with low-dose colistin against a diverse collection of Gram-negative bacterial pathogens. *Eur. J. Clin. Microbiol. Infect. Dis.* 32 1291–1294. 10.1007/s10096-013-1875-z 23609511

[B28] Pushpakom S. Iorio F. Eyers P. A. Escott K. J. Hopper S. Wells A. (2019). Drug repurposing: progress, challenges and recommendations. *Nat. Rev. Drug Discov.* 18:41. 10.1038/nrd.2018.168 30310233

[B29] Roberts K. D. Azad M. A. Wang J. Horne A. S. Thompson P. E. Nation R. L. (2015). Antimicrobial activity and toxicity of the major lipopeptide components of polymyxin B and colistin: last-line antibiotics against multidrug-resistant Gram-negative bacteria. *ACS Infect. Dis.* 1 568–575. 10.1021/acsinfecdis.5b00085 27525307 PMC4980087

[B30] Santajit S. Indrawattana N. (2016). Mechanisms of antimicrobial resistance in ESKAPE pathogens. *BioMed Res. Int.* 2016:2475067. 10.1155/2016/2475067 PMC4871955 27274985

[B31] Tamma P. D. Cosgrove S. E. Maragakis L. L. (2012). Combination therapy for treatment of infections with Gram-negative bacteria. *Clin. Microbiol. Rev.* 25:450. 10.1128/cmr.05041-11 22763634 PMC3416487

[B32] Theuretzbacher U. Gottwalt S. Beyer P. Butler M. Czaplewski L. Lienhardt C. (2019). Analysis of the clinical antibacterial and antituberculosis pipeline. *Lancet Infect. Dis.* 19 e40–e50. 10.1016/s1473-3099(18)30513-9 30337260

[B33] Velkov T. Thompson P. E. Nation R. L. Li J. (2010). Structure–activity relationships of polymyxin antibiotics. *J. Med. Chem.* 53 1898–1916. 10.1021/jm900999h 19874036 PMC2907661

[B34] Venter H. (2019). Reversing resistance to counter antimicrobial resistance in the World Health Organisation’s critical priority of most dangerous pathogens. *Biosci. Rep.* 39:BSR20180474. 10.1042/BSR20180474 PMC6465202 30910848

[B35] Venter H. Shilling R. A. Velamakanni S. Balakrishnan L. Van Veen H. W. (2003). An ABC transporter with a secondary-active multidrug translocator domain. *Nature* 426 866–870. 10.1038/nature02173 14685244

[B36] Ventola C. L. (2015). The antibiotic resistance crisis: part 1: causes and threats. *Pharm. Ther.* 40 277–283. PMC4378521 25859123

[B37] Wang Y. Alenzy R. Song D. Liu X. Teng Y. Mowla R. (2020). Structural optimization of natural product nordihydroguaretic acid to discover novel analogues as AcrB inhibitors. *Eur. J. Med. Chem.* 186:111910. 10.1016/j.ejmech.2019.111910 31801655

[B38] Willyard C. (2017). The drug-resistant bacteria that pose the greatest health threats. *Nature* 543:15. 10.1038/nature.2017.21550 28252092

[B39] Woolhouse M. Farrar J. (2014). Policy: an intergovernmental panel on antimicrobial resistance. *Nat. News* 509:555. 10.1038/509555a 24877180

[B40] World Health Organization [WHO] (2019). *WHO Advisory Group on Integrated Surveillance of Antimicrobial Resistance (AGISAR): Critically Important Antimicrobials for Human Medicine 6th Revision 2018.* Geneva: WHO.

[B41] Zilberberg M. D. Shorr A. F. Micek S. T. Vazquez-Guillamet C. Kollef M. H. (2014). Multi-drug resistance, inappropriate initial antibiotic therapy and mortality in Gram-negative severe sepsis and septic shock: a retrospective cohort study. *Crit. Care* 18:596. 10.1186/s13054-014-0596-8 PMC4264255 25412897

