# Supplementary material for: In vitro Activity of Robenidine Analog NCL195 in Combination With Outer Membrane Permeabilizers Against Gram-Negative Bacterial Pathogens and Impact on Systemic Gram-Positive Bacterial Infection in Mice
Source: Front Microbiol. 2020 Aug 4;11:1556. doi: 10.3389/fmicb.2020.01556 (PMC7417630; doi:10.3389/fmicb.2020.01556)
Supplement: Supplementary file 1 [file Data_Sheet_1.pdf]

Supplementary Material

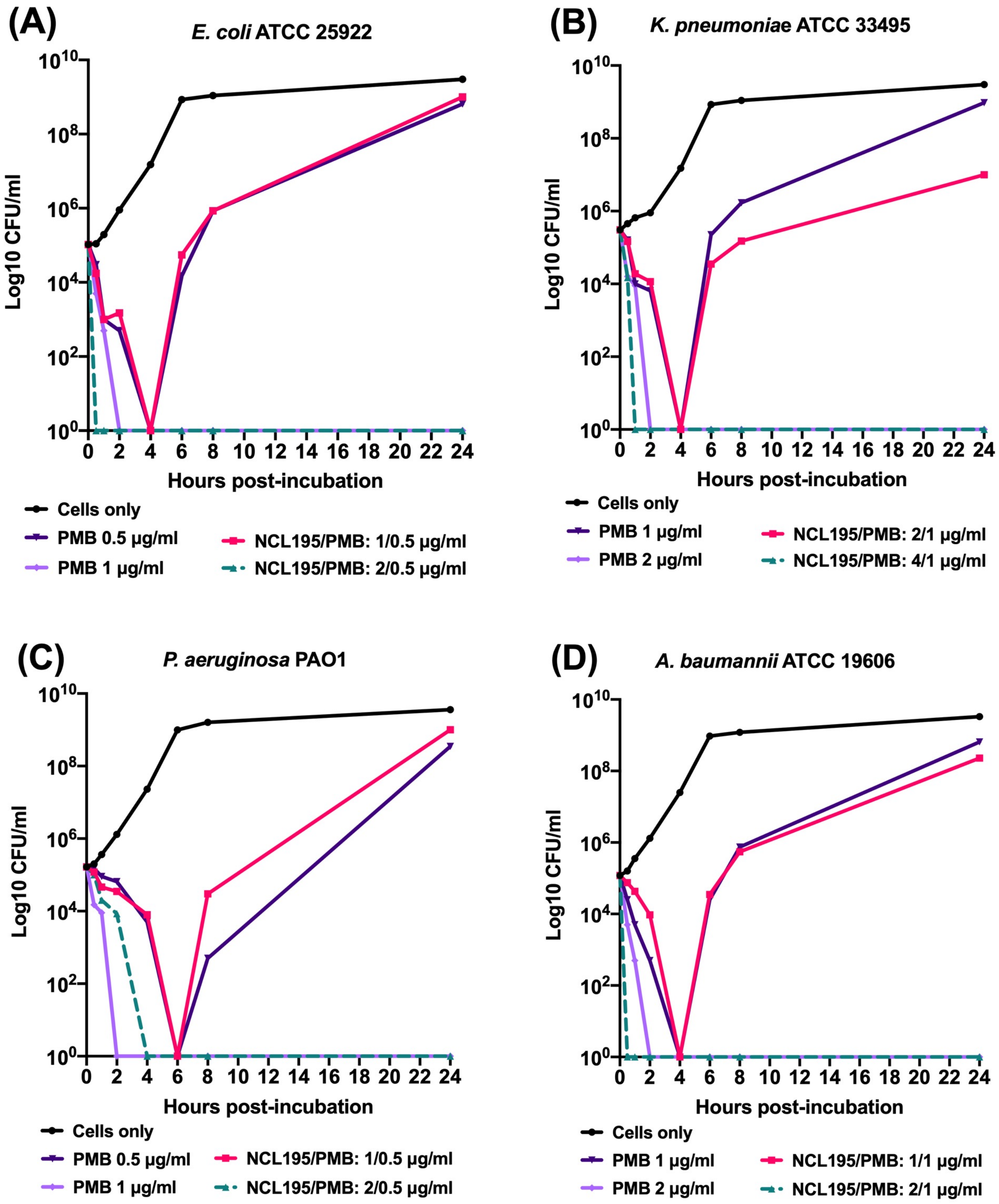

Figure S1. Time kill curves of NCL195 and PMB combinations against *E. coli* ATCC 25922 (**A**), *K. pneumoniae* ATCC 33495 (**B**), *P. aeruginosa* PAO1(**C**) and *A. baumannii* ATCC 19606 (**D**).
